# Effect of taurine supplementation on preventing ventilator-associated pneumonia in pediatrics under mechanical ventilation, a randomized controlled double-blind clinical trial

**DOI:** 10.3389/fped.2024.1490247

**Published:** 2025-01-10

**Authors:** Nasrin Shirzad-Yazdi, Eslam Shorafa, Seyedeh Narjes Abootalebi, Reza Heidari, Katayoon Hojabri, Marziyeh Doostfatemeh, Fatemeh Masjedi, Afsaneh Vazin, Mojtaba Shafiekhani

**Affiliations:** ^1^ Student Research Committee, Shiraz University of Medical Sciences, Shiraz, Iran; ^2^ Department of Clinical Pharmacy, School of Pharmacy, Shiraz University of Medical Sciences, Shiraz, Iran; ^3^ Department of Pediatrics, Division of Intensive Care Unit, School of Medicine, Shiraz University of Medical Sciences, Shiraz, Iran; ^4^ Biotechnology Research Center, Shiraz University of Medical Sciences, Shiraz, Iran; ^5^ Pharmaceutical Sciences Research Center, Shiraz University of Medical Sciences, Shiraz, Iran; ^6^ Department of Biostatistics, School of Medicine, Shiraz University of Medical Sciences, Shiraz, Iran; ^7^ Nephro-Urology Research Center, Shiraz University of Medical Sciences, Shiraz, Iran

**Keywords:** pediatric intensive care unit, septic shock, taurine, ventilator-associated pneumonia, pneumonia

## Abstract

**Introduction:**

One of the most prevalent healthcare-associated infections in the pediatric intensive care unit is ventilator-associated pneumonia (VAP). VAP not only results in prolonged hospital and intensive care unit (ICU) stays but also imposes higher costs on patients and the healthcare system. Therefore, it is essential to implement preventive measures. The lung-protective properties of taurine are recognized, and this research focuses on assessing the impact of taurine supplementation in preventing VAP.

**Method:**

This double-blind, randomized clinical trial was conducted at Namazi Hospital's PICUs. The study included pediatrics on mechanical ventilation for over 48 h. Patients were randomly divided into two groups: the taurine and placebo groups. Alongside the standard care, participants from both groups were administered taurine or placebo capsules (30 mg/kg. day) in divided doses from the day of PICU admission through PICU discharge. The incidence of VAP through clinical and laboratory evidence was considered the primary outcome.

**Results:**

Seventy-seven patients were included in the study, with 38 in the taurine group and 39 in the placebo group. VAP incidence was 7.9% in the taurine group and 64.1% in the placebo group. Taurine significantly reduced the duration of mechanical ventilation, ICU and hospital stay, and inotrope duration. The occurrence of septic shock was lower in the taurine group at 5.3%. Stepwise logistic regression showed that placebo receipt was the only risk factor for VAP, with placebo recipients being 20.8 times more likely to develop VAP. (*P* < 0.0001, OR 20.8, 95% CI 6.11–97.93) Taurine treatment also significantly reduced inflammatory markers such as CRP, pro-calcitonin, and interleukin-6 compared to placebo.

**Conclusion:**

Our results showed that taurine supplementation can reduce the incidence of VAP and the duration of mechanical ventilation, ICU, and hospital stay in critically ill pediatric patients.

**Registration number of the clinical trial:**

This study received approval from the Iranian registry clinical trial, registered on 29 June 2023 (IRCT20120731010453N4, http://www.irct.ir/).

## 1 Introduction

Mechanical ventilation (MV) is often necessary for critically ill pediatrics in intensive care, but it can lead to complications, such as ventilator-associated pneumonia (VAP). VAP is the second most common infection in pediatric intensive care units (PICUs) and has a high mortality rate of 33% to 71% (1, 2). A study on pediatric cardiac surgery patients in India found that VAP increased the duration of mechanical ventilation by 3.7 days, resulting in an estimated cost of $11,897 per episode (3).

Various interventions have been studied and proven effective in reducing the incidence of VAP in adult intensive care units (ICUs), but their impact on ventilated pediatric patients is not well-researched. The most commonly used interventions and recommendations in pediatrics include hand hygiene, mouth care with an antiseptic solution, and elevating the head of the bed by 30 to 45 degrees (4–6). Despite these interventions, the incidence of VAP remains high, particularly in developing countries (7). It is crucial to explore novel pharmacological and non-pharmacological interventions to reduce the incidence of VAP and its associated complications.

Taurine, a β-amino acid naturally present in various animal tissues, particularly excitable tissues like the brain and muscle, plays crucial roles in several physiological processes (8).

While taurine is an essential nutrient for certain animals, it is considered conditionally essential in humans. The human body synthesizes taurine in limited quantities, and overt deficiency is rarely observed (9).

Unlike adults, infants cannot produce taurine on their own and rely on the taurine provided by their mothers through the placenta or breast milk (10). In infants, taurine plays various roles in the central nervous system, contributing to both development and neuroprotection (11).

A taurine-rich diet is essential to fulfill their dietary requirements and address any deficiencies, particularly for infants who cannot be breastfed for various reasons and need taurine through their diet (12). Zamboni G et al. demonstrated that low dietary taurine intake can hinder vitamin D absorption in preterm infants, suggesting that taurine supplementation in preterm infant formulas is advisable (13). Although there is limited research on the effects of taurine in pediatric patients, one older study indicated that taurine supplementation helped reduce the loss of fatty acids from conjugated bile acids in cystic fibrosis patients with elevated glycine-to-taurine ratios (14). Additionally, finding that taurine-supplemented PN provided significant protective benefits against parenteral nutrition-associated cholestasis (PNAC) in premature infants or those with necrotizing enterocolitis (NEC). This supports the connection between taurine deficiency and health issues (15).

Many research has revealed taurine's vital functions, particularly in modulating immune responses and mitigating inflammatory diseases. Studies indicate that taurine and its derivatives can reduce inflammatory cell infiltration and cytokine production in various experimental models (16). Taurine plays a crucial role in nutrition, particularly in its effectiveness in treating mitochondrial diseases, including mitochondrial encephalopathy, lactic acidosis, and stroke-like episodes (MELAS). Additionally, it presents a promising approach for managing metabolic disorders like diabetes and inflammatory conditions such as arthritis. In the central nervous system, taurine's multifaceted functions include supporting developmental processes and offering cellular protection (9, 17). Sun et al. found that taurine supplementation led to a reduction in blood pressure through enhanced flow- and nitroglycerin-mediated dilation, which was not seen in the placebo group. The taurine-treated participants also showed increased plasma taurine and H2S levels, with H2S contributing to lower blood pressure by inhibiting TRPC3-induced signaling in blood vessels (18). Its antioxidant properties are vital in neutralizing harmful substances and preventing mitochondrial damage, which significantly contributes to energy metabolism and cellular signaling (19).

Despite its high concentrations in tissues such as the lungs, spleen, and reproductive organs, the specific functions of taurine in these systems remain largely unexplored. Preliminary studies in humans suggest taurine's osmoregulatory properties may play a critical role in these organs (10). in cases of sepsis-induced lung injury, taurine has been shown to enhance the protective effects of dexmedetomidine by inhibiting the Nuclear factor kappa B (NF-κB) signaling pathway and lowering levels of inflammatory molecules such as Interleukin 6 (IL-6) and Interleukin 1β (IL-1β) (20–22). Additionally, taurine aids in preserving epithelial integrity by enhancing the expression of epithelial cadherin and occludin, thereby alleviating lung injury in rat models subjected to cecal ligation and puncture (23).

Due to the promising therapeutic effects of taurine, the aim of this study was to evaluate its potential role in preventing VAP among critically ill pediatrics.

## 2 Method

### 2.1 Study design

This parallel, randomized, controlled double-blind clinical study was conducted at Namazi Hospital in southern Iran. Namazi Hospital is the largest tertiary educational referral center in the south Iran. It is associated with the Shiraz University of Medical Sciences, Shiraz, Iran. This research was carried out in a PICU comprising 18 beds. Three pediatric intensive care specialists and two clinical pharmacists visited and assessed the patients. Eligible patients were included from June 2023 to March 2024. The patients were randomly assigned to either the intervention or placebo group in a 1:1 ratio using a blocked randomization technique overseen by an independent biostatistician. Our study randomized samples in groups of 4 patients (50 blocks). This study received approval from the Clinical Ethics Committee of Shiraz University of Medical Sciences (IR.SUMS.REC.1401.385), and it has also been registered in the Iranian registry clinical trial (IRCT20120731010453N4 http://www.irct.ir/). All protocols adhered to the ethical guidelines outlined in the 1975 Helsinki Declaration (24).

Before enrolling in the research, the participants' guardians received details about the study's objectives. Each participant's parents were required to fill out an informed consent form. Participants were entitled to exit the survey at any time without prior notification.

### 2.2 Sample size estimation

Based on data from previously published studies (6), and with a study power of 1- *β* = 0.8 and *α* = 0.05, along with a 1:1 allocation ratio between the treatment and placebo groups, it was estimated that a total of 33 participants would be needed in each group. Taking into account a projected attrition rate of 10%, the sample size required was determined to be a minimum of 36 patients per group.

### 2.3 Study precipitants

Pediatrics aged three months to 15 years who had been on mechanical ventilation for more than 48 h were included in the study. However, pediatrics with solid or hematologic malignancy, heart failure, premature, expired within the first 72 h of hospitalization, and Cystic fibrosis (CF) were excluded from the study. They are visited every day by a critical care specialist and clinical pharmacist for clinical and interventions.

### 2.4 Interventions

Taurine (ALLMAX ® Company, Canada) and Carboxy Methyl Cellulose (CMC) as placebo (MERCK ® Company, Germany) were produced in the pharmaceutical laboratory of Shiraz Faculty of Pharmacy in the dosage form of capsules. Both groups received a daily dose of 30 mg/kg, taurine or CMC administered orally in divided doses every 12 h from the study's involvement until discharge from the PICUs. The capsules were opened, dissolved in water per the prescribed dose, and given to the patients through an NG tube. To maintain the confidentiality of the assignments, the capsules were made to look the same in terms of shape, size, and color and were packaged in identical containers.

Each container had a code that indicated whether it contained taurine or CMC. Still, this information was only known to an independent technician and remained anonymous until the end of the study.

### 2.5 Blinding

As mentioned before, this study was designed as a double-blind, placebo-controlled clinical trial. This means that all potential participants including patients, their families, physicians, and clinical pharmacists were kept unaware of the treatment assignments. This approach helps to minimize any selection bias.

### 2.6 VAP diagnosis

The pediatric critical care specialist evaluated all patients daily for incidence of VAP. The diagnosis of VAP was made according to the Center for Disease Control and Prevention criteria (CDC) (25).

The initial diagnosis is based on clinical suspicion and specific criteria on two or more serial chest radiographs. Additionally, mini-bronchoalveolar lavage (mini-BAL) was utilized for the identification of isolated pathogens. (New or progressive radiographic infiltrates, consolidation, cavitation, and pneumatoceles in an infant ≤1).

For pediatrics older than one year or up to 12 years old, criteria include fever (>38. 0°C ^1^or > 100. 4°F ^2^) or hypothermia (<36. 0°C or <96.8°F), leukopenia(≤4,000 WBC ^3^/mm^3^ ^4^) or leukocytosis(≥15,000 WBC/mm^3^), new onset of purulent sputum or increased respiratory secretions, worsening cough or dyspnea, rales or bronchial breath sounds, and worsening gas exchange(for example, O_2_^5^ desaturations [for example, pulse oximetry <94%], increased oxygen requirements, or increased ventilator demand).

For pediatrics older than 12 years old, criteria include Fever (>38.0°C or >100.4°F), Leukopenia (≤4,000 WBC/mm^3^) or leukocytosis (≥12,000 WBC/mm^3^), And the new onset of purulent sputum or change in character of sputum, or increased respiratory secretions, or increased suctioning requirements Dyspnea, or tachypnea, or new onset or worsening cough, Rales or bronchial breath sounds Worsening gas exchange [for example, O_2_ desaturations (for instance, PaO_2_ ^6^/FiO_2_ ^7^ ≤ 240), increased oxygen requirements, or increased ventilator demand]For infants under one year old, alternate criteria include temperature instability, leukopenia(≤4,000 WBC/mm^3^) or leukocytosis(≥15,000 WBC/mm^3^) with left shift(≥10% band forms), new onset of purulent sputum or increased respiratory secretions, apnea or tachypnea, wheezing or rales, cough, and bradycardia(<100 beats/min) or tachycardia(>170 beats/min) (25).

### 2.7 Primary and secondary outcomes

The primary outcome of this study was to compare the incidence of VAP between the groups receiving a placebo or taurine during mechanical ventilation from the day of PICU admission through PICU discharge. Additional outcomes assessed included the occurrence of sepsis, septic shock, usage of inotropes and vasopressors, duration of mechanical ventilation, length of stay in the ICU and hospital, and mortality rate.

### 2.8 Data gathering

The demographic and para-clinical data, including age, sex, weight, past medical and drug history, diagnosis at admission, reasons for mechanical ventilation, complete cell count, BUN, serum creatinine, and the Pediatric Index of Mortality-3 (PIM- 3) scores, were recorded for each patient at baseline. Prospective monitoring was conducted to track the duration of mechanical ventilation, length of ICU and hospital stay, the incidence of VAP, septic shock, mortality rates, and signs and symptoms of infection (such as temperature, leukocyte counts, differential counts, interpretation of chest radiographs, blood, sputum, and mini BAL culture results). All medications, such as antibiotics and inotropes, with dose and duration, were also closely monitored and documented throughout the study.

### 2.9 Blood sampling and inflammatory biomarker assays

Blood samples 5 milliliter (ml) were collected at each specified time interval (at the beginning and end of the PICU stay) in standard gel-coated tubes (Vacutest® Kima, Italy) to prepare serum. For this purpose, blood samples were centrifuged (3,000 *g*, 10 min, 4°C) and the supernatants were then stored at −70°C until assessing inflammatory biomarkers. (IL-6) and IL-1B levels were measured using the Human enzyme-linked immunoassay (ELISA) kit (Karmania-Pars Gene®, IRAN, CN: KPG-HIL6, KPG-HIL1β). Additionally, the serum levels of CRP and pro-calcitonin were determined using the immunoturbidimetric method (BioSystem Kit®, Spain). ELISA measurements of IL-6 and IL-1B were conducted based on the kit's instructions (Karmania-Pars Gene®, IRAN). Briefly, 100 *μ*l of standards or samples were added to the appropriate wells (in triplicates). The plate was covered and incubated at room temperature on an orbital shaker for one hour. Then, the wells were emptied and washed (at least five times) with wash buffer and 100 *μ*l of reconstituted anti-cytokines biotin conjugates were added. The plate was then covered and incubated for one hour (room temperature, orbital shaker). Afterward, the plate was emptied again, and washed five times with washing buffer, and 100 *μ*l of streptavidin-HRP working solution was added and incubated for 30 min (room temperature, orbital shaker). Finally, the plate was emptied, washed (five times), and 100 *μ*l of the substrate solution was added. The plate was covered and incubated at room temperature on an orbital shaker and the absorbance of the developed color was measured at *λ* = 650 using a BioTek® multifunctional plate reader. The concentration of IL-6 and IL- IL-1β was calculated using a standard curve.

### 2.10 Follow up

As mentioned VAP is definitively confirmed only when patients present both clinical symptoms and radiographic changes, in addition to a positive culture from mini-bronchoalveolar lavage (mini-BAL). Preventive measures, including hand washing and raising the head of the bed to an angle of 30–45 degrees, were implemented for all patients. Daily assessments were conducted to monitor for signs of ventilator-associated pneumonia (VAP) throughout the study. If there was clinical suspicion of VAP, mini-BAL, complete blood counts, and blood cultures, pro-calcitonin, and CRP levels were performed. Those diagnosed with VAP received antibiotic therapy following standard guidelines. The typical duration of antibiotic treatment for VAP was seven days for patients who responded clinically.

### 2.11 Statistical analysis

Mean and standard deviation (SD) were calculated for quantitative variables. While factors without a normal distribution were described using median and interquartile range (IQR). Bivariate analysis was conducted using independent sample *T*-test and chi-square test, followed by multivariate analysis via stepwise logistic regression to identify factors associated with the outcome variable. The multivariate logistic regression model included all variables with a *p*-value less than 0.20 in the bivariate analysis. A *p*-value of less than 0.05 was considered statistically significant, and odds ratios (ORs) with a 95% confidence interval (CI) were reported. Furthermore, to determine the extent of the impact of taurine on secondary outcomes, Cohen's effect size was classified as small (ES = |0.2|), medium (ES = |0.5|), and large (ES = |0.8|) (26).

Statistical analyses, including the receiver operating characteristic (ROC) curve, were performed using Blue-sky software, version 10.3.2. (Grayslake, IL, USA).

## 3 Result

### 3.1 Enrollment and demographic data

**Figure 1:**
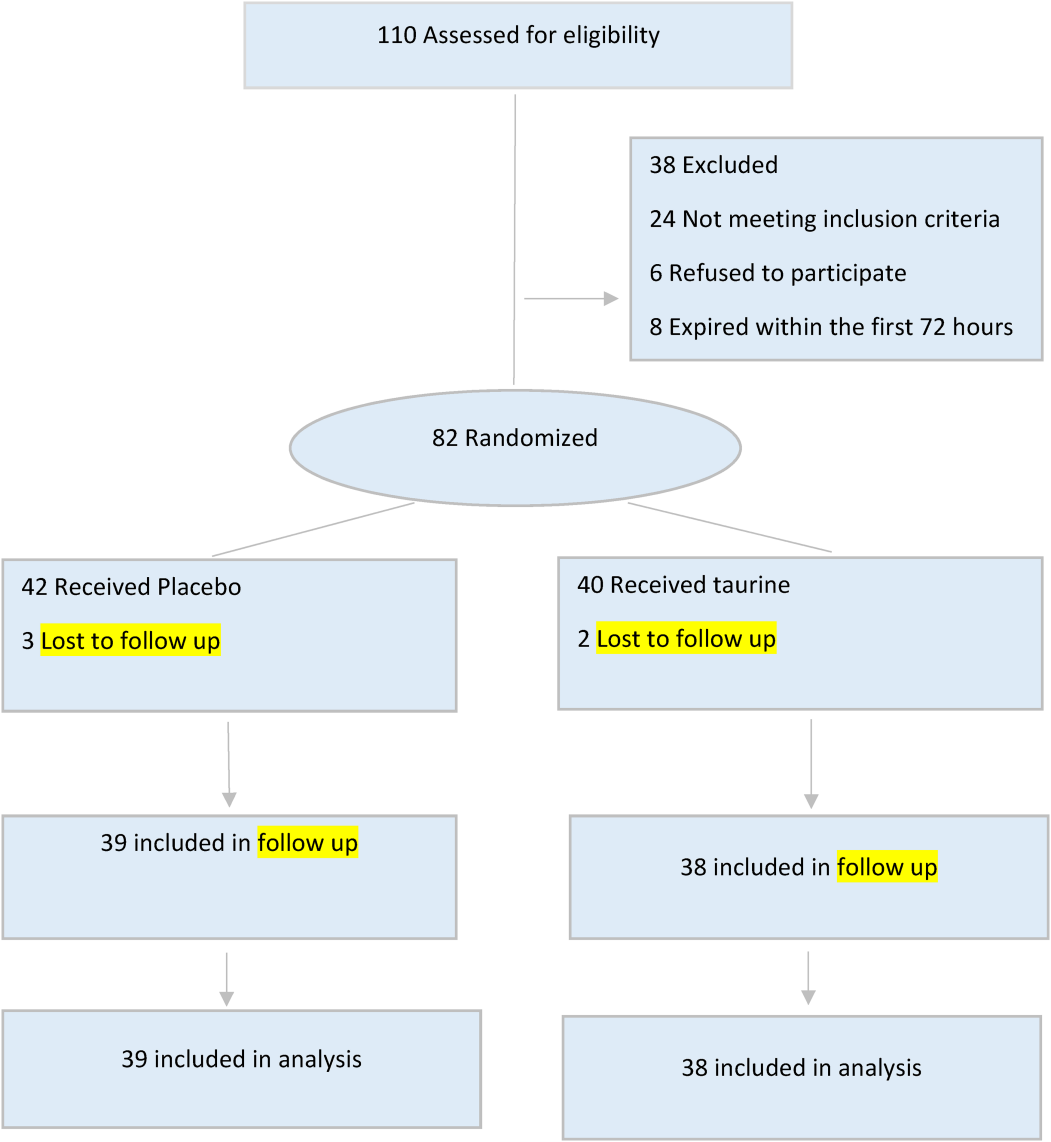
CONSORT flow diagram of the study.

During the study period, 110 pediatrics who required mechanical ventilation were admitted to the PICU. Out of these, 77 pediatrics met the criteria for inclusion in the study. Among the 77 pediatrics, 38 received taurine, while 39 received a placebo. A flow diagram (Figure 1) summarizes the patient recruitment and randomization process. Boys comprised 47.3% of the taurine group (*n* = 39) and 58.9% of the placebo group (*n* = 28). The mean age of all participants was 63.19 ± 61.60 months, with the taurine group having a mean age of 68.10 ± 59.98 months and the placebo group having a mean age of 58.41 ± 53.55 months, Neurological disorders were the most common cause of admission and underlying disease among the participants. The demographic and clinical data for both groups are shown in Table 1.

**Table 1:** Baseline demographic and clinical data among pediatrics admitted to PICU (*N* = 77).

Variables		Total (*N* = 77)	Taurine (*N* = 38)	Placebo (*N* = 39)	*P*-value
Age, mean ± SD^a^ (month)		63.19 ± 61.60	68.10 ± 59.98	58.41 ± 53.55	0.61
PIM3 score^b^, mean ± SD		5.26 ± 7.17	4.51 ± 8.27	6.00 ± 5.93	0.36
Wight (Kg), mean ± SD		20.76 ± 18.02	20.06 ± 14.81	21.43 ± 20.85	0.74
Sex, *n* (%)	Girl	36 (46.75)	20 (52.63)	16 (41.02)	0.30
	Boy	41 (53.24)	18 (47.36)	23 (58.97)	
Underlying diseases *n* (%)	No	42 (54.54)	19 (50)	23 (58.97)	0.56
	Neurologic disorder	14 (18.18)	8 (21.05)	6 (15.38)	
	Hematologic disorder	6 (18.7)	2 (5.26)	4 (10.25)	
	Metabolic disease	3 (3.89)	1 (2.63)	2 (5.12)	
	Others^k^	12 (15.58)	8 (21.05)	4 (10.25)	
Causes of admission *n* (%)	Neurologic disorder	40 (51.94)	21 (55.26)	19 (48.71)	0.48
	Poisoning	11 (14.28)	4 (10.52)	7 (17.94)	
	Upper respiratory tract Infection	10 (12.98)	4 (10.52)	6 (15.38)	
	Cardiovascular disease	5 (6.49)	2 (5.26)	3 (7.69)	
	Metabolic disease	3 (3.89)	3 (7.89)	0	
	Others^l^	8 (10.38)	4 (10.52)	4 (10.25)	
BUN^c^ (mg/dl), mean ± SD		11.83 ± 8.86	10.34 ± 6.33	13.28 ± 10.66	0.14
Serum Cr^d^,(mg/dl), mean ± SD,		0.53 ± 0.24	0.54 ± 0.25	0.52 ± 0.24	0.73
Cr Cl^e^, ml/min, (modified Schwartz), mean ± SD		112.02 ± 48.45	114.03 ± 47.27	110.05 ± 50.1	0.72
WBC^f^ (×1,000 mm^3^)		9,459.70 ± 3,892.81	9,465.79 ± 3,684.53	9,453.84 ± 4,133.03	0.98
CRP^g^ (mg/L)		30.07 ± 35.2	33.13 ± 34.55	27.10 ± 36.03	0.45
PCT^h^ (ng/L)		1.5 ± 3.2	1.60 ± 3.80	1.45 ± 2.59	0.83
IL-6^i^ (pg/ml)		3.2 ± 0.48	3.45 ± 0.46	2.97 ± 0.37	<0.001
IL-1 β^j^ (pg/ml)		185.6 ± 45.6	184.46 ± 54.30	186.90 ± 36.74	0.90

^a^ Standard Deviation.

^b^ Pediatric Index of Mortality 3.

^c^ Blood urea nitrogen.

^d^ Serum Creatinine.

^e^ Creatinine Clearance.

^f^ white blood cells.

^g^ C-reactive protein.

^h^ Pro-calcitonin.

^i^ Interleukin-6.

^j^ Interleukin-1 beta.

^k^ Others include = Two cases of asthma, Five cases of G6PD, One case of renal stone, One case of migraine, Three cases of Diabetes Mellitus.

^l^ Others include = Three cases of chicken Pox, Two cases of gastro-intestinal bleeding, One case of Autoimmune Hepatitis, Two cases of Acute liver failure.

### 3.2 Clinical outcome

#### 3.2.1 Primary outcome

During the study period, 28 pediatrics (36.4%) were diagnosed with VAP. Three pediatrics (7.9%) in the taurine groups had confirmed VAP, as compared with 25 of the (64.9%) pediatrics in the placebo group (*P* < 0.0001). The VAP incidence rate was 50.5/1.000 ventilator days.

#### 3.2.2 Secondary outcome

The two study groups had a significant statistical difference regarding the length of mechanical ventilation day, ICU, and hospital stay. The median length of mechanical ventilation in the taurine group was four days (IQR = 7), compared to the placebo group was ten days (IQR = 15) (*P* = 0.001). The median length of ICU stay in the taurine group was 8.5 days (IQR = 10.5), compared to the placebo group was 14 days (IQR = 22) (*P* = 0.003). The median hospital stay in the taurine group was 11.5 days (IQR = 10.75), compared to the placebo group, was 19days (IQR = 26.5), (*P* = 0.004).

Furthermore, among the secondary outcomes, the impact of taurine on the length of mechanical ventilation demonstrated a significant effect with a large effect size. (ES = |0.8|) Additionally, it exhibited medium effect sizes on the length of ICU stay, (ES = |0.7|) hospital stay, (ES = |0.67|) as well as the duration of inotrope and vasopressor use, (ES = |0.52|).

The occurrence of septic shock was 10.4%; this in the taurine group was 5.3%, vs. 15.4% in the placebo group. (*P* = 0.23), Furthermore, The mean norepinephrine equivalent dose (27), in the taurine and placebo group was 0.12 ± 0.14 (*µ*g/kg/min) and 0.24 ± 0.5 (*µ*g/kg/min), respectively (*p* = 0.08). The median length of inotrope and vasopressor duration was three days (IQR = 6.5) in the taurine group, while in the placebo group, it was 5.5 days (IQR = 5.2), with statistical significance (*P* = 0.02). Eighteen pediatrics died during the study: seven (18.4%) in the taurine group and eleven (28.2%) in the placebo group (Table 2), (*p* = 0.40). No side effects related to the intervention were observed in pediatrics throughout the follow-up period. More details are shown in Table 2.

**Table 2:** Comparison of clinical outcomes between placebo and taurine groups in mechanical ventilation pediatrics (*N* = 77).

Variables		Total (*N* = 77)	Taurine (*N* = 38)	Placebo (*N* = 39)	*P*-value	Effect size
VAP^a^ *n* (%)	Yes	28 (36.4)	3 (7.9)	25 (64.1)	**<0** **.** **001**	
	No	49 (63.6)	35 (92.1)	14 (35.9)		
Mortality *n* (%)	Yes	18 (23.4)	7 (18.4)	11 (28.2)	0.4207	
	No	59 (76.6)	31 (81.6)	28 (71.8)		
Inotrope and vasopressor administration *n* (%)	Yes	58 (75.3)	26 (68.4)	32 (82.1)	0.1942	
	No	19 (24.7)	12 (31.6)	7 (17.9)		
Septic Shock *n* (%)	Yes	8 (10.4)	2 (5.3)	6 (15.4)	0.2626	
	No	69 (89.6)	36 (94.7)	33 (84.6)		
Norepinephrine equivalent dose, mean ± SD^b^		0.18 ± 0.37	0.12 ± 0.14	0.24 ± 0.5	0.08	
Length of inotrope and vasopressor duration (Days) Median, (IQR)^c^		5 (6)	3 (6.5)	5.5 (5.2)	**0** **.** **02**	0.52
Length of mechanical duration (Days) Median, (IQR)		7 (10)	4 (7)	10 (15)	**0** **.** **001**	0.80
Length of ICU stay (days) Median, (IQR)		11 (12)	8.5 (10.5)	14 (22)	**0** **.** **003**	0.70
Length of hospital stay (Days) Median, (IQR)		16 (14)	11.5 (10.75)	19 (26.5)	**0** **.** **004**	0.67

Variables with a *P*-value of less than 0.05 are highlighted in bold.

^a^ Ventilator Associated Pneumonia.

^b^ Standard Deviation.

^c^ Interquartile Range.

#### 3.2.3 Laboratory and microbiological outcome

**Figure 2:**
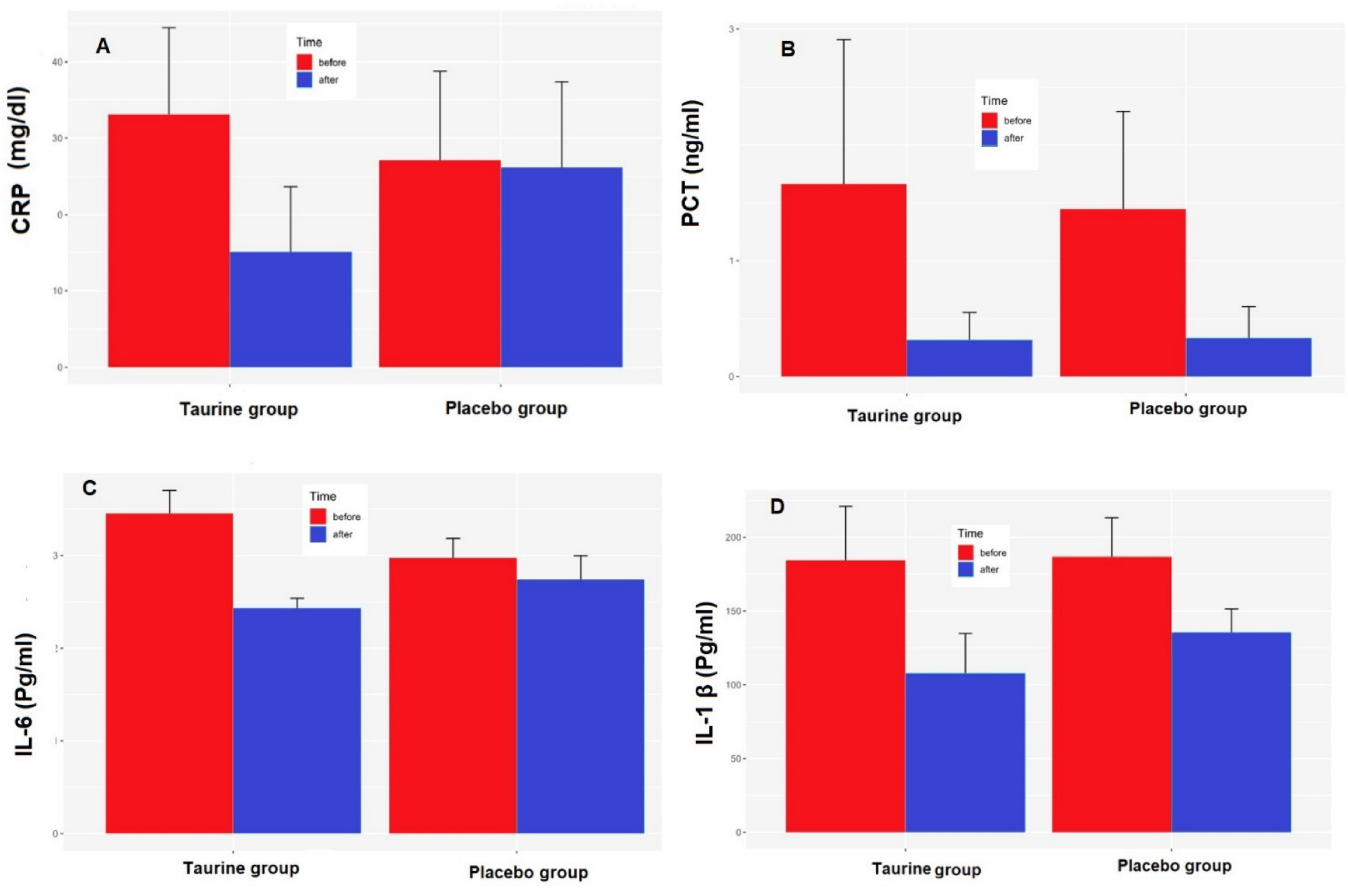
Effects of taurine on **(A)** C-reactive protein (CRP), **(B)** Pro-calcitonin (PCT), **(C)** serum interleukin 6 (IL-6), **(D)** serum interleukin 1-Beta (IL-1β), data were present as mean ± SE for taurine (*n* = 38) and placebo (*n* = 39), *P* < 0.05 for between-group comparisonsgroups.*P < 0.05 for between-group comparisons.

At the onset of the clinical trial, there were no significant differences in CRP, PCT, and IL-1 β levels between the taurine and placebo groups (*p* > 0.05). (Table 1) After calculating mean differences, a significant statistical contrast emerged between the taurine and placebo groups concerning CRP (*P* < 0.04) and IL-6 levels (*P* < 0.0001). The reduction in CRP, PCT, and IL-1 β levels was notably more pronounced in the taurine group compared to the placebo group, though not statistically significant (Figure 2).

The most isolated pathogens responsible for VAP were gram-negative species such as Acinetobacter baumannii, NFB, and Klebsiella pneumonia, which were often found to be poly-microbial (20.5%). There were significant differences in the distribution of pathogen types between the VAP and non-VAP groups (*P* < 0.001). Pathogens isolated from the lower respiratory tract of patients with VAP are reported in Table 3.

**Table 3:** Types of microorganisms from mini-BAL in pediatrics with VAP (*N* = 28).

Type of isolated pathogen	Taurine group (*N* = 3)	Placebo group (*N* = 25)	*P*-value
Acinetobacter baumannii	1 (33.3)	1 (4)	*P* < 0.001
NFB^a^	-	2 (8)	
Klebsiella pneumonia	-	1 (4)	
Stenotrophomonas maltophilia	-	2 (8)	
Streptococcus species	-	2 (8)	
Candida species	-	2 (8)	
Poly-microbial (Acinetobacter baumannii + NFB, pseudomonas aeruginosa + Candida Albicans, Acinetobacter baumannii + pseudomonas aeruginosa + NFB)	-	8 (32)	

^a^ Non fermentative bacilli, Values are *n* (%).

### 3.3 Risk factors for VAP

The study examined various factors that contribute to the incidence of VAP. These factors included age, sex, PIM3-score, reintubation, administration of corticosteroids and Proton-pump inhibitors (PPI), prior antibiotic therapy, enteral nutrition, presence of bloodstream infection, and the number of inotropes/vasopressors. Binary logistic regression with stepwise (Likelihood Ratio) was applied. The accuracy of the predictive model was 80%; assessed by the area under the ROC curve. The odds of having VAP were about 20.8 [OR = 20.8, 95% CI: (6.11–97.93)] times higher in the placebo group than in the taurine group. (*P* value **<** **0.001**), (Table 4).

**Table 4:** Factor associated with VAP based on the stepwise logistic regression model.

Variables	B^a^	Std. Error^b^	OR^c^, (95% CI^d^)	*p*-value
Placebo	3.03	0.68	20.8 (6.11–97.93)	<0.001

^a^ Coefficient β.

^b^ Standard Error.

^c^ Odds Ratio.

^d^ Confident Interval.

## 4 Discussion

VAP is a frequent issue among patients on mechanical ventilation (28, 29). This clinical trial was conducted to investigate the prophylactic effect of taurine on the incidence of VAP in pediatrics. Our results show that taurine can be considered a promising agent for preventing VAP.

In our study, we observed that 25.4% of patients developed VAP. Based on a recent review article in 2022, pediatric studies worldwide have reported a variable incidence of VAP ranging from 2%–35% among mechanically ventilated patients in PICUs (5). Discrepancies in patient profiles, nutritional status, sample sizes, PICU vs. NICU, available resources, and diagnostic criteria could explain these differences.

VAP is associated with increased rates of morbidity, death, antibiotic consumption, and medical costs (30, 31). Also, VAP can lengthen hospitalizations by up to two days and raise the risk of death by as much as 14%, especially in those with pre-existing health issues (13). Consequently, various pharmacological and non-pharmacological strategies have been implemented to mitigate the incidence of VAP. Several studies have explored the effectiveness of VAP bundle prevention strategies such as head elevation, oral hygiene, sedation cessation, and open and closed suctioning (32–34). While research in infant and pediatric populations is limited, evidence indicates that implementing a combination of care measures can successfully lower rates of VAP (35–37). However, in a study conducted by Osman et al. in Saudi Arabia, the impact of a VAP prevention package was examined before and after implementation in the PICU. The study's results showed that the VAP bundle did not result in a statistically significant reduction in VAP rates in the PICU (1). So, pharmacological interventions to prevent VAP are the focus of attention today. A recent randomized controlled trial conducted by Roshanzamiri et al. has shown that daily use of a probiotic containing Limosilactobacillus reuteri during hospitalization in the PICU reduced the incidence of VAP in the probiotic group compared to the placebo group significantly (44.12% VS 21.05%) (6). Our research had even more significant results than this study regarding VAP incidence, duration of mechanical ventilation days, length of ICU and hospital stay, and duration of inotrope. Although probiotics are used in many fields and have favorable effects, there are reports of increased chances of sepsis, bacteremia, and fungemia and increased length of hospital stay, especially in critically ill pediatrics under two years of age (38–40). Hence, more studies are needed on the safety of probiotics therapy.

Probiotics diminish the colonization of pathogen microorganisms using local and systemic interventions (41, 42), whereas taurine's mechanism operates intracellularly (21). It is essential to mention that previous studies on the potential protective effects of taurine against lung damage have mainly been conducted on animals. Therefore, it isn't easy to understand the exact mechanism of taurine's function. In a study by Tapan M Bhavsar et al., Syrian hamsters with LPS-induced lung inflammation used a single dose of taurine (50 mg/kg/day). They demonstrated protective properties against inflammatory, oxidative, and apoptotic effects caused by bacterial endotoxin (43). Another study by Camila de Oliveira Ramos et al. found that consuming taurine could reduce inflammation and oxidative stress in the lungs of adult mice exposed to cigarette smoke for a short period (44). Moreover, a report indicates that administering taurine and curcumin to rats can effectively alleviate lung tissue damage caused by bisphenol A, including problems like bleeding, atrophy, and emphysema (45).

In sepsis-induced lung injury, it is crucial to recognize the importance of inflammatory signaling pathways, specifically the Mitogen-activated protein kinases (MAPK) and NF-κB pathways, in the excessive release of inflammatory cytokines like Tumor necrosis factor alpha (TNF-α) and IL-1β. These pathways play a significant role in contributing to lung dysfunction and mortality in sepsis-induced lung injury (46–48). A study by Jiao Chen *et al*. demonstrated the potential of taurine in alleviating sepsis-induced lung injury by inhibiting the inflammatory response and oxidative stress by suppressing the p38/MAPK signaling pathway (8). Furthermore, research has highlighted the role of IL-1β in acute lung injury through integrin-dependent mechanisms (49), with patients with VAP experiencing elevated levels of pro-inflammatory mediators.

In this regard, in a recent study by Tu Liang Liang et al., taurine was found to play a dual role in lung cancer progression in immune-competent mice for the first time. The research suggests that taurine may affect the tumor immune response via the Nfe2l1-ROS-PD-1 signaling pathway. There is a strong correlation between Nfe2l1 and key components of the taurine metabolism pathway. The effects of taurine on lung cancer progression vary depending on immune competence, with Nfe2l1 exhibiting anti-tumor properties. In immune-deficient mice, taurine may exert anti-tumor effects by inhibiting NF-κB-mediated inflammatory responses.

IL-6, a cytokine that promotes inflammation and fibrosis, is pivotal in developing respiratory diseases such as asthma, COPD^8^, and IPF^9^ (50, 51). Uncontrolled levels of IL-6 can worsen lung inflammation and contribute to the progression of respiratory disorders. While current therapeutic approaches involve IL-6-neutralizing antibodies to manage inflammation and autoimmunity, these treatments can be costly (52). In this regard, taurine has been shown to bind to TLR4 and inhibit the TLR4/NF-κB pathway, ultimately reducing pro-inflammatory factors (TNF-α, and IL-6) and oxidative stress. These findings highlight the essential role of taurine in maintaining intestinal barrier integrity and inhibiting intestinal inflammation, suggesting that taurine is a promising supplement for the treatment of colitis (53). Another study showed that Tau-Cl inhibition of IL-6 and IL-8 synthesis in FLS from RA patients is due to the ability of this compound to reduce the activity of key transcriptional regulators (NF-kB and AP-1), which subsequently reduces the transcription of these (54).

In line with previous research, our findings indicate that taurine successfully decreases biomarkers associated with inflammation, including CRP, pro-calcitonin, IL-6, and IL-1β, particularly in reducing levels of IL-6 and CRP (55–57). A study by L. O. Mallasiy further supports our findings, showing similar reductions in CRP and IL-6 among participants receiving taurine (58).

Overall, the results suggest that taurine has potential as a therapeutic agent for reducing the inflammatory response and oxidative stress associated with sepsis-induced lung injury, providing potential benefits for patients at risk of lung dysfunction and mortality in this context. Moreover, a trial conducted among type 2 diabetes mellitus patients demonstrated that supplementation with 1,000 mg of taurine for eight weeks positively affected oxidative stress status (59), aligning with the results of the research above.

`Various factors in mechanically ventilated patients weaken the immune system and increase the risk of VAP, including critical illness, comorbidities (60), malnutrition (61), decreased cough reflex due to endotracheal intubation (62), and direct access for bacteria to move from the upper to the lower respiratory tract (63, 64). The combination of impaired host defense and continuous exposure to potential pathogens through the endotracheal tube makes these patients vulnerable to VAP (65). Our findings show that taurine administration can reduce the length of mechanical ventilation and decrease the incidence of VAP compared to a placebo. In contrast to a previous study where patients were ventilated for an average of 9 days, our study found an average duration of 4 days (6). Furthermore, research has indicated that patients with VAP experience more extended periods of ventilation and stay in the ICU compared to those without VAP (66).

Interestingly, pediatrics who received taurine had a shorter stay in the ICU. Our results are consistent with a study by Motaghi et al., who used taurine for post-transplant adult patients (67). In contrast, Roshan Zamiri et al. reported that the length of hospitalization in the intensive care unit and hospital is longer, and their outcomes are not statistically significant (6).

Our research revealed a significant difference between the taurine and placebo groups' average duration of inotrope and vasopressor use. Additionally, the incidence of septic shock was found to be three times higher in the placebo group compared to the drug group. These results underscore the importance of taurine in critical illness. Taurine regulates cell volume and addresses osmotic imbalances (68). It also possesses antioxidant and anti-inflammatory properties, potentially reducing sepsis and membrane damage from ischemia/reperfusion (16, 69). Mitochondrial function heavily relies on taurine, and its deficiency can lead to respiratory chain dysfunction and increased oxidative stress (70). Vermeulen *et al*. demonstrated a decrease in taurine levels in critically ill patients with septic shock, which may compromise cellular protection against inflammation and oxidative stress, ultimately contributing to cellular damage (71). As explained above, the reduction in septic shock incidence observed in the PICU presents encouraging findings that warrant further exploration and investigation.

Another interesting finding of our study was that taurine can reduce the risk of pathogenic bacteria. This finding is consistent with the favorable immunomodulatory and antimicrobial effects of taurine or taurolidine, which have been reported in several experimental studies (72–74).

It is important to note that the intervention in this study did not result in any negative effects. Taurine has been deemed safe by the Food and Agriculture Organization of the United Nations (FAO)/WHO^10^ and is generally regarded as a safe (GRAS) agent. Research in children has demonstrated taurine to be safe and beneficial (75, 76). Studies on taurine in premature infants have also shown no adverse reactions (77).

Our study, being the first of its kind, holds significant importance in highlighting the potential preventive effects of taurine on the incidence of VAP in children. However, this study had several limitations: One of the key limitations of this study is the small sample size, which restricts the ability to draw definitive conclusions and accurately measure the incidence rate of VAP. Data from this study were obtained from a single center, which may introduce inherent biases. The study did not assess 28-day mortality rates. Additionally, a fixed dose of taurine was utilized throughout the study, suggesting that further research is needed to determine the optimal minimum drug dosage for maximum efficacy. Future studies should investigate varying dosages to understand the potential impact on outcomes better.

## 5 Conclusion

Taurine can significantly contribute to preventing VAP. Reducing the duration of ventilation, staying in the ICU and hospitalization, reducing the use of inotrope, and reducing the incidence of septic shock are among the other remarkable achievements of this research. These findings indicate that taurine supplementation may hold promise as a potential intervention for reducing VAP incidence.

## Data Availability

The original contributions presented in the study are included in the article/Supplementary Material, further inquiries can be directed to the corresponding authors.

## Footnotes

^1^Centigrade

^2^Fahrenheit

^3^White Blood Cells

^4^cubic millimeter

^5^oxygen

^6^Partial pressure of oxygen

^7^Fraction of inspired oxygen

^8^Chronic obstructive pulmonary disease

^9^Idiopathic pulmonary fibrosis

^10^World Health Organization
